# Supplementary material for: Linkage-based ortholog refinement in bacterial pangenomes with CLARC
Source: Nucleic Acids Res. 2025 Jun 20;53(12):gkaf488. doi: 10.1093/nar/gkaf488 (PMC12204703; doi:10.1093/nar/gkaf488)
Supplement: gkaf488_Supplemental_Files [file gkaf488_supplemental_files.zip › CLARC_supplementary_R1.pdf]

SUPPLEMENTARY INFORMATION

Linkage-based ortholog refinement in bacterial pangenomes with CLARC

Indra González Ojeda[1,3], Samantha G. Palace[1,2], Pamela P. Martinez[4], Taj Azarian[5], Lindsay R. Grant[6], Laura L. Hammitt[6], William P. Hanage[1], Marc Lipsitch[1,2]

[1] Center for Communicable Disease Dynamics, Department of Epidemiology, T.H. Chan School of Public Health, Harvard University, Boston, Massachusetts, United States of America

[2] Department of Immunology and Infectious Diseases, T.H. Chan School of Public Health, Harvard University, Boston, Massachusetts, United States of America

[3] Harvard Biophysics Graduate Program, Graduate School of Arts and Sciences, Harvard University, Boston, Massachusetts, United States of America

[4] Department of Microbiology, University of Illinois Urbana-Champaign, Champaign, Illinois, United States of America

[5] Burnett School of Biomedical Sciences, University of Central Florida, Orlando, Florida, United States of America

[6] Department of International Health, Johns Hopkins Bloomberg School of Public Health, Baltimore, Maryland, United States of America

SUPPLEMENTARY TABLES

Table S1. CLARC clusters found in pangenomes generated from the Southwest, USA dataset + 9 closed references (946 genomes) using different tools and parameters.

Pangenome tool/parameters	Number of accessory CLARC clusters	Number of core CLARC clusters	Total # of CLARC clusters
Roary i98	269	139	**408**
Roary, i95 (default)	99	21	**120**
Roary, i90	95	24	**119**
Roary, i80	89	27	**116**
PPanGGOLiN i98	211	58	**269**
PPanGGOLiN i95	62	18	**80**
PPanGGOLiN i90	46	14	**60**
PPanGGOLiN i80 (default)	26	14	**40**
Panaroo, i98 – strict (default)	8	1	**9**
Panaroo, i98 – moderate	8	2	**10**
Panaroo, i98 – sensitive	9	2	**11**
Panaroo, i95 - strict	3	1	**4**
Panaroo, i95 - moderate	7	1	**8**
Panaroo i95 - sensitive	7	1	**8**
Panaroo, i90 – strict	2	1	**3**
Panaroo, i90 – moderate	3	2	**5**
Panaroo, i90 – sensitive	3	2	**5**
Panaroo, i80 – strict	2	0	**2**
Panaroo, i80 – moderate	3	1	**4**
Panaroo, i80 – sensitive	3	1	**4**

Table S2. CLARC clusters found in pangenomes generated from the samples in all carriage datasets + 9 closed references (8907 genomes) using different tools and parameters.

Pangenome tool/parameters	Number of accessory CLARC clusters	Number of core CLARC clusters	Total # of CLARC clusters
Roary, i98	401	479	**880**
Roary, i95 (default)	274	453	**727**
Roary, i90	247	452	**699**
Roary, i80	217	430	**647**
PPanGGOLiN i98	180	62	**242**
PPanGGOLiN i95	66	22	**88**
PPanGGOLiN i90	40	15	**55**
PPanGGOLiN i80 (default)	26	10	**36**
Panaroo, i98 – strict (default)	2	0	**2**
Panaroo, i98 – moderate	3	0	**3**
Panaroo, i98 – sensitive	5	0	**5**
Panaroo, i95 - strict	2	0	**2**
Panaroo, i95 - moderate	6	0	**6**
Panaroo, i95 - sensitive	1	0	**1**
Panaroo, i90 – strict	1	0	**1**
Panaroo, i90 – moderate	3	0	**3**
Panaroo, i90 – sensitive	0	0	**0**
Panaroo, i80 – strict	2	0	**2**
Panaroo, i80 – moderate	2	1	**3**
Panaroo, i80 – sensitive	1	1	**2**

Table S3. CLARC reduction of the accessory genome/increase of the core genome in pangenome analyses generated using pangenome analyses from different bacterial species.

Pangenome tool/parameters	Species	Post-CLARC accessory gene change	Post-CLARC Core gene change
Roary, i95 (default)	*M. tuberculosis*	-97	+26
Roary, i90	*M. tuberculosis*	-70	+19
Roary, i80	*M. tuberculosis*	-75	+19
PPanGGOLiN i95	*M. tuberculosis*	-71	+31
PPanGGOLiN i90	*M. tuberculosis*	-74	+33
PPanGGOLiN i80 (default)	*M. tuberculosis*	-51	+23
Panaroo, i98 – strict (default)	*M. tuberculosis*	0	0
Panaroo, i90 – strict	*M. tuberculosis*	0	0
Panaroo, i80 – strict	*M. tuberculosis*	0	0
Roary, i95 (default)	*L. monocytogenes*	-82	+9
Roary, i90	*L. monocytogenes*	-31	+4
Roary, i80	*L. monocytogenes*	-25	+2
PPanGGOLiN i95	*L. monocytogenes*	-55	+5
PPanGGOLiN i90	*L. monocytogenes*	-21	+2
PPanGGOLiN i80 (default)	*L. monocytogenes*	-15	+3
Panaroo, i98 – strict (default)	*L. monocytogenes*	-1	0
Panaroo, i90 – strict	*L. monocytogenes*	-1	0
Panaroo, i80 – strict	*L. monocytogenes*	-3	0
Roary, i95 (default)	*S. agalactiae*	-76	+10
Roary, i90	*S. agalactiae*	-62	+6

Roary, i80	*S. agalactiae*	-63	+6
PPanGGOLiN i95	*S. agalactiae*	-73	+15
PPanGGOLiN i90	*S. agalactiae*	-49	+10
PPanGGOLiN i80 (default)	*S. agalactiae*	-41	+8
Panaroo, i98 – strict (default)	*S. agalactiae*	-7	0
Panaroo, i90 – strict	*S. agalactiae*	-8	0
Panaroo, i80 – strict	*S. agalactiae*	-6	0
Roary, i95 (default)	*S. pneumoniae*	-115	+14
Roary, i90	*S. pneumoniae*	-89	+5
Roary, i80	*S. pneumoniae*	-84	+6
PPanGGOLiN i95	*S. pneumoniae*	-131	+31
PPanGGOLiN i90	*S. pneumoniae*	-84	+19
PPanGGOLiN i80 (default)	*S. pneumoniae*	-65	+15
Panaroo, i98 – strict (default)	*S. pneumoniae*	-8	1
Panaroo, i90 – strict	*S. pneumoniae*	-4	1
Panaroo, i80 – strict	*S. pneumoniae*	-3	0
Roary, i95 (default)	*E. coli*	-358	+34
Roary, i90	*E. coli*	-293	+18
Roary, i80	*E. coli*	-299	+17
PPanGGOLiN i95	*E. coli*	-115	+13
PPanGGOLiN i90	*E. coli*	-62	+8
PPanGGOLiN i80 (default)	*E. coli*	-41	+6
Panaroo, i98 – strict (default)	*E. coli*	-10	0
Panaroo, i90 – strict	*E. coli*	-5	0
Panaroo, i80 – strict	*E. coli*	-5	0

Table S4. CLARC reduction of the accessory genome/increase of the core genome in pangenome analyses generated with bacterial samples encompassing multiple species within the same genus. Genus level datasets for *Brucella*, *Chlamydia*, *Klebsiella* and *Enterococcus* were the same as those used in the RIBAP manuscript[1]. The dataset for *Streptococcus* contained a total of 76 closed genomes. Half of those genomes (38 genomes) correspond to *Streptococcus pneumoniae* and the other half to *Streptococcus agalactiae*. Accession numbers for all samples used in these analyses can be found in the **Additional file 2** excel sheet.

Pangenome tool/parameters	Genus	Post-CLARC accessory gene change	Post-CLARC Core gene change
Roary, i95 (default)	*Brucella*	-126	+22
Roary, i90	*Brucella*	-99	+8
Roary, i80	*Brucella*	-98	+8
PPanGGOLiN i95	*Brucella*	-142	+57
PPanGGOLiN i90	*Brucella*	-97	+37
PPanGGOLiN i80 (default)	*Brucella*	-79	+32
Panaroo, i98 – strict (default)	*Brucella*	0	0
Panaroo, i90 – strict	*Brucella*	0	0
Panaroo, i80 – strict	*Brucella*	0	0
Roary, i95 (default)	*Chlamydia*	-19	0
Roary, i90	*Chlamydia*	-3	0
Roary, i80	*Chlamydia*	-2	0
PPanGGOLiN i95	*Chlamydia*	-22	0

PPanGGOLiN i90	*Chlamydia*	-10	0
PPanGGOLiN i80 (default)	*Chlamydia*	-7	0
Panaroo, i98 – strict (default)	*Chlamydia*	-1	0
Panaroo, i90 – strict	*Chlamydia*	-1	0
Panaroo, i80 – strict	*Chlamydia*	0	0
Roary, i95 (default)	*Klebsiella*	-91	+2
Roary, i90	*Klebsiella*	-61	0
Roary, i80	*Klebsiella*	-67	+1
PPanGGOLiN i95	*Klebsiella*	-40	+1
PPanGGOLiN i90	*Klebsiella*	-40	+1
PPanGGOLiN i80 (default)	*Klebsiella*	-11	+1
Panaroo, i98 – strict (default)	*Klebsiella*	-5	0
Panaroo, i90 – strict	*Klebsiella*	-3	0
Panaroo, i80 – strict	*Klebsiella*	-4	0
Roary, i95 (default)	*Enterococcus*	-70	0
Roary, i90	*Enterococcus*	-57	0
Roary, i80	*Enterococcus*	-52	0
PPanGGOLiN i95	*Enterococcus*	-52	0
PPanGGOLiN i90	*Enterococcus*	-35	0
PPanGGOLiN i80 (default)	*Enterococcus*	-23	0
Panaroo, i98 – strict (default)	*Enterococcus*	-8	0
Panaroo, i90 – strict	*Enterococcus*	-8	0
Panaroo, i80 – strict	*Enterococcus*	-8	0
Roary, i95 (default)	*Streptococcus*	-90	+1
Roary, i90	*Streptococcus*	-57	0
Roary, i80	*Streptococcus*	-72	0
PPanGGOLiN i95	*Streptococcus*	-127	+1
PPanGGOLiN i90	*Streptococcus*	-87	0
PPanGGOLiN i80 (default)	*Streptococcus*	-77	0
Panaroo, i98 – strict (default)	*Streptococcus*	-11	0
Panaroo, i90 – strict	*Streptococcus*	-13	0
Panaroo, i80 – strict	*Streptococcus*	-10	0

SUPPLEMENTARY FIGURES

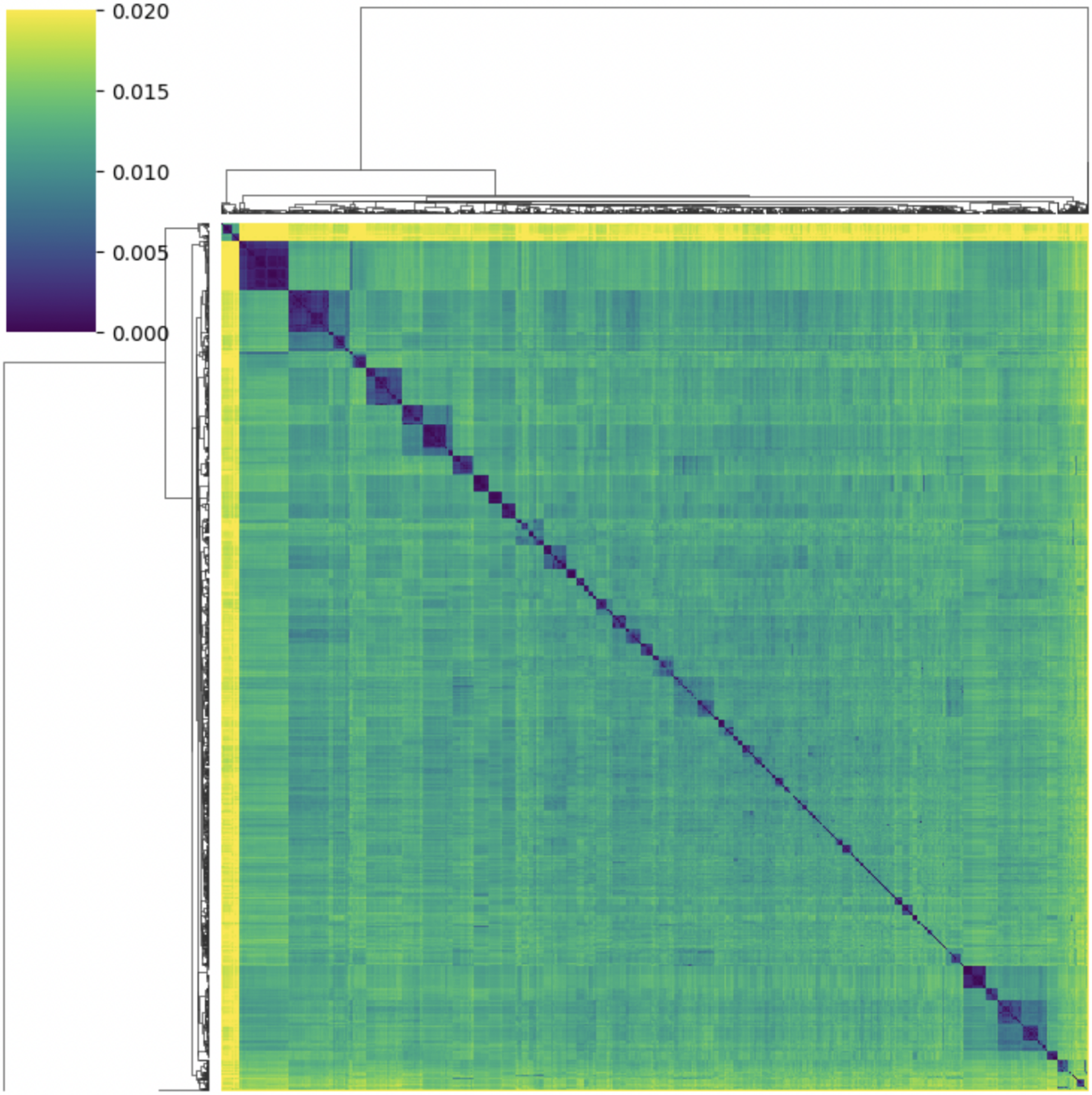

Figure S1. *Mash distances for all 8,907 S. pneumoniae genomes used in this study.* Average genomic distance is 1.24%. Mash was run on default parameters.

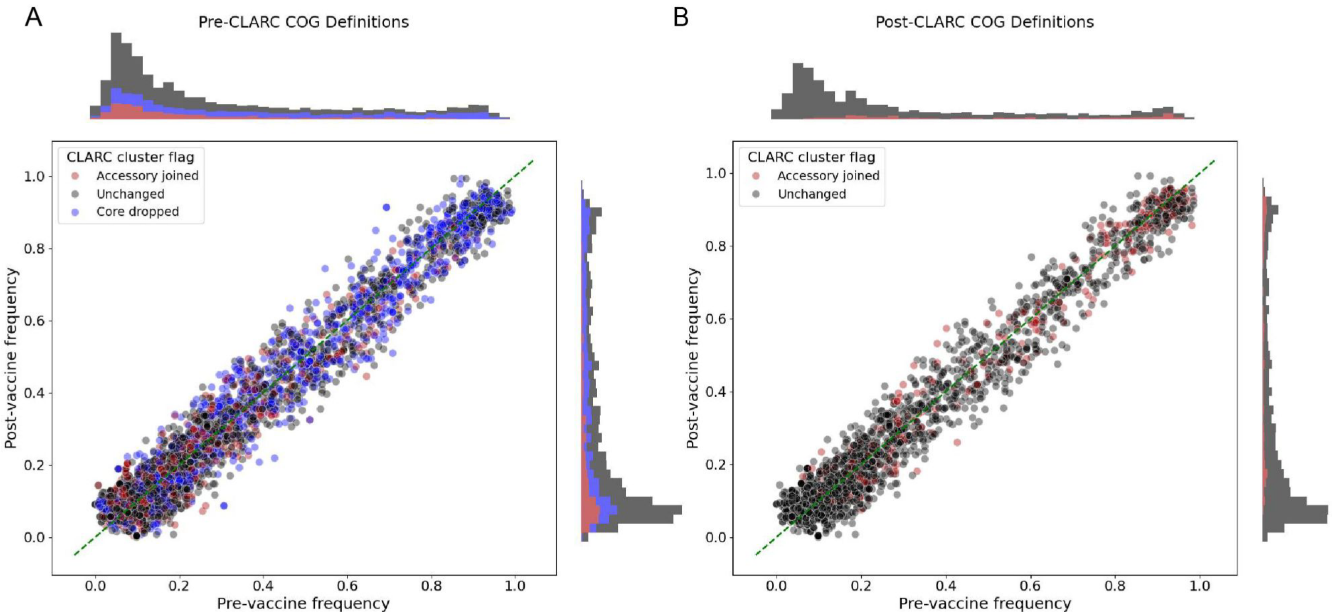

Figure S2. *Effect of CLARC in the pre- and post-vaccination frequency of accessory genes in the Southwest US population.* **(A)** Using pre-CLARC gene definitions and **(B)** using post-CLARC accessory gene definitions. COGs identified as part of a CLARC cluster are colored. Blue dots represent COGs identified as part of a cluster that turned out to be a core gene when condensed, while red dots represent COGs that were part of a cluster that remained an accessory gene after CLARC correction. In **(B)** the blue dots don't appear because they were eliminated from the set of accessory genes (since they were re-classified as core genes), but the new COGs representing condensed accessory gene clusters are marked in red. The frequencies plotted for the post-CLARC genes are the condensed frequencies of these new COGs.

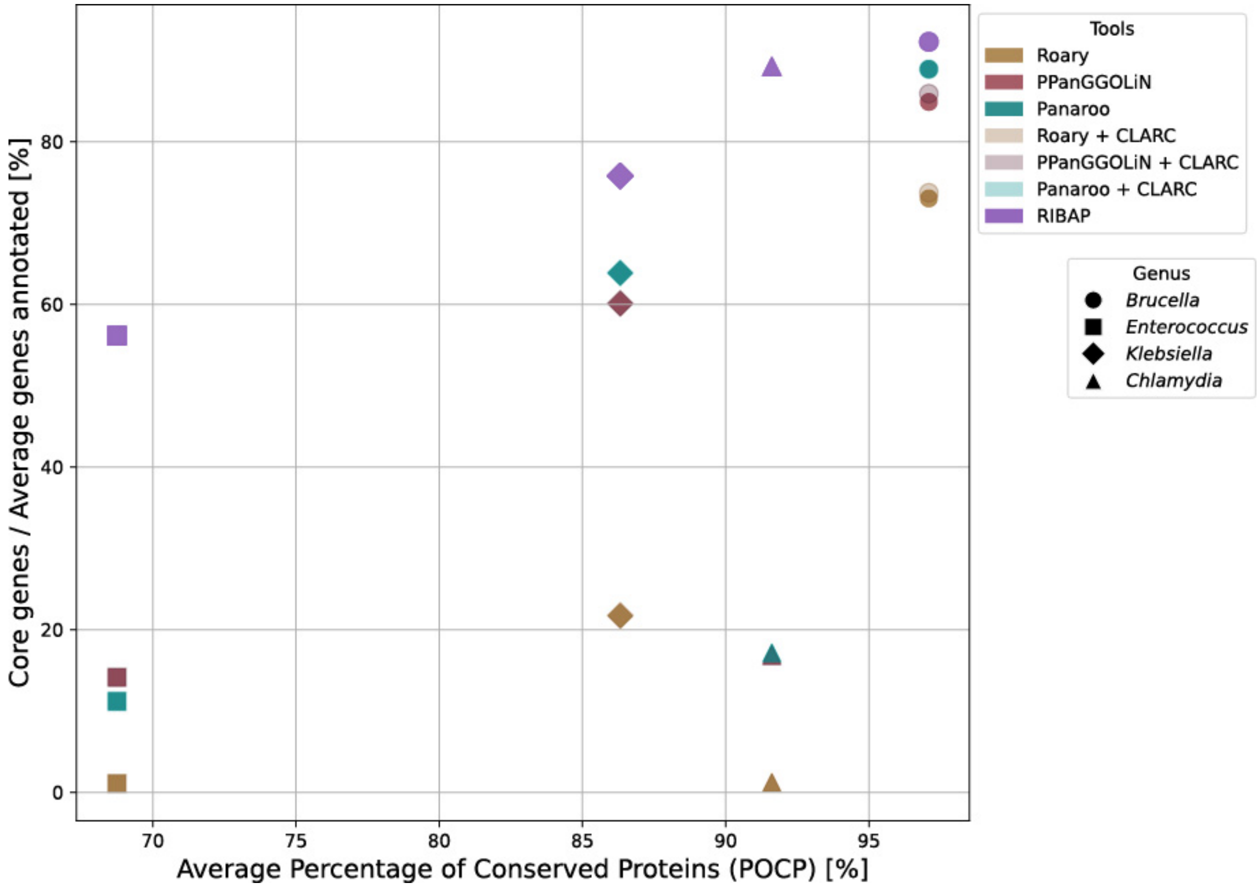

Figure S3. *Comparison of genus-level ortholog refinement of the core genome with RIBAP vs CLARC (v1.2.0)*. RIBAP results and average percentage of conserved protein (POCP) values were obtained from the original RIBAP manuscript's supplementary data[1], specifically document "13059_2024_3312_MOESM4_ESM.xlsx". Core genes here are defined as those COGs present in >95% of all samples.

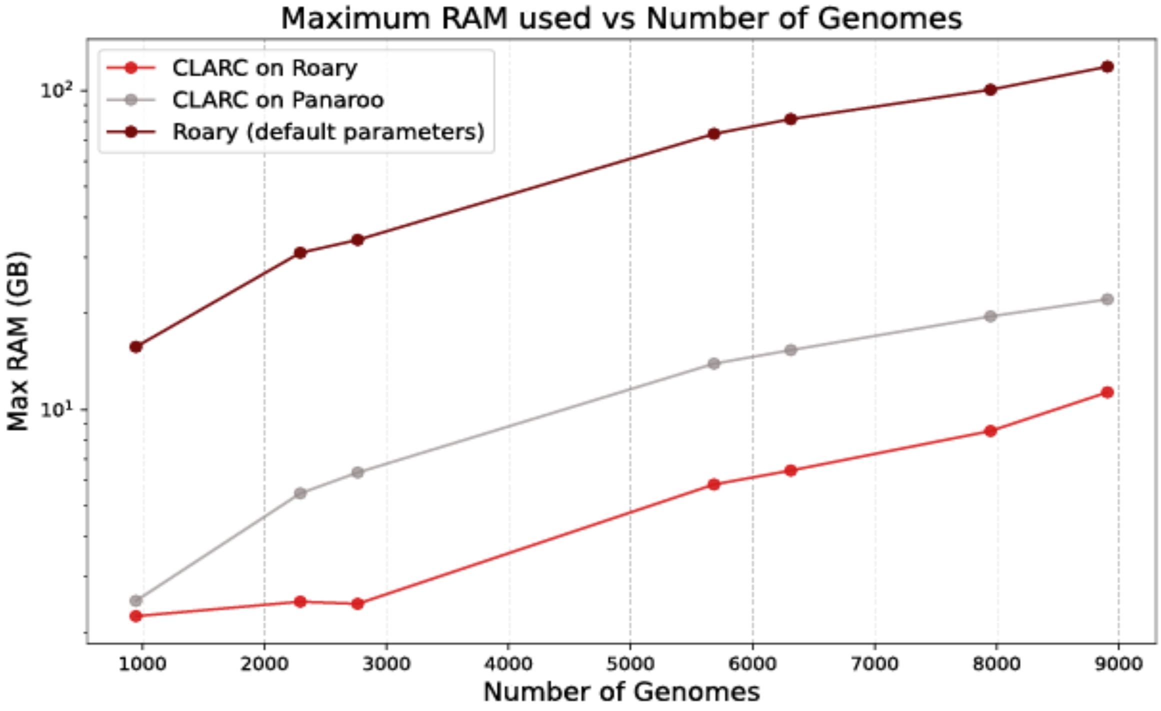

Figure S4. *Computational performance of CLARC* (v1.1.0). Performance for Panaroo is not pictured because running Panaroo on all pneumococcal genomes in one job required more computational resources than available in the FASRC computing cluster. All multi-dataset Panaroo analyses shown in the main text were obtained through a 2-step process where we ran Panaroo individually on each carriage dataset, and then ran the appropriate merge commands within Panaroo to obtain the pangenome definitions of runs with >1,000 genomes.

REFERENCES

1. Lamkiewicz, K., Barf, L.-M., Sachse, K. & Hölzer, M. RIBAP: a comprehensive bacterial core genome annotation pipeline for pangenome calculation beyond the species level. *Genome Biol.* **25**, 170 (2024).
